# Management Challenges of Metastatic Spinal Cord Compression in Pregnancy

**DOI:** 10.1155/2020/8891021

**Published:** 2020-11-01

**Authors:** Davor Dasic, Narendra K. Rath, Mario Ganau, Zaid Sarsam

**Affiliations:** ^1^Department of Neurosurgery, The Walton Centre NHS Foundation Trust, Liverpool, UK; ^2^Department of Neurosurgery, Oxford University Hospitals NHS Foundation Trust, Oxford, UK

## Abstract

Primary and secondary spinal tumours with cord compression often represent a challenging condition for the patient and clinicians alike, even more so during pregnancy. The balance between safe delivery of a healthy baby and management of the mother's disease bears many clinical, psychological, and ethical dilemmas. Pregnancy sets a conflict between the optimal surgical and oncological managements of the mother's tumour and the well-being of her foetus. We followed the CARE guidelines from the EQUATOR Network to report an exemplificative case of a 39-year-old woman with a 10-year history of breast cancer, presenting in the second trimester of her first pregnancy with acute onset severe thoracic spinal instability, causing mechanical pain and weakness in lower limbs. Neuroradiological investigations revealed multilevel spinal deposits with a pathological T10 fracture responsible for spinal cord compression. The patient was adamant that she wanted a continuation of the pregnancy and her baby delivered. After discussion with her oncologist and obstetrician, we agreed to perform emergency spinal surgery—decompression and instrumented fixation. The literature search did not reveal a similar case of spinal metastatic breast cancer undergoing spinal instrumentation and delivery of a healthy baby a few months later. Following the delivery, the patient had further oncological treatment, including chemotherapy and radiotherapy. The paucity of such reports prompted us to present this case and highlight the relevance of a multidisciplinary approach involving obstetrician, oncologist, spinal surgeon, and radiologist to guide the optimal decision-making process.

## 1. Introduction

Cancer during pregnancy is rare and accounts between 0.02 and 0.1% of all pregnancy [1]; nonetheless, it represents the second most common cause of death in the reproductive years after heart disease [2]. With rising gestational age, now peaking in the late 30s and early 40s in the developed countries, the incidence of malignancy during pregnancy is expected to keep on increasing [3]. This occurrence represents a significant challenge for the patient, family, and the medical teams. The balance between pregnancy management, delivery of a baby, and the coordinated strategies for oncological and surgical management poses many professional and ethical questions. At the same time, achieving this balance also claims a psychological burden on the patient and her carers, including all healthcare professionals involved. These multifaceted issues make the case for a comprehensive multidisciplinary approach; nonetheless, the paucity of information available on the literature limits the ability of such management strategy to provide evidence-based care. For this reason, we followed the CARE guidelines from the EQUATOR Network to report an exemplificative case and discuss all the educational points that would be of benefit to our community.

## 2. Case Report

We present a case of a 39-year-old female who was referred to the Walton Centre in Liverpool with a 10-year history of breast cancer (ER-positive (7/8), PgR-positive (8/8), and HER2 FISH-negative invasive ductal carcinoma). Previously, she had left mastectomy and axillary clearance, prophylactic right mastectomy, and followed by a course of chemotherapy (FEC/docetaxel) and radiotherapy (40 Gy) of the left chest wall and supraclavicular fossa. In addition, she received five years of adjuvant tamoxifen.

**Figure 1:**
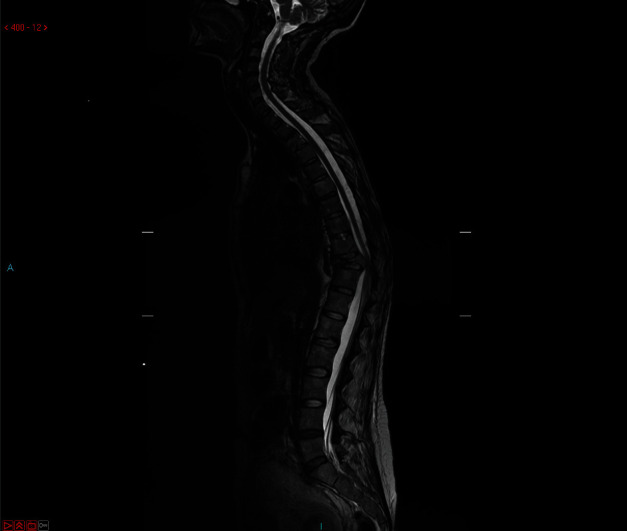
Preoperative MRI T2WI (whole spine).

**Figure 2:**
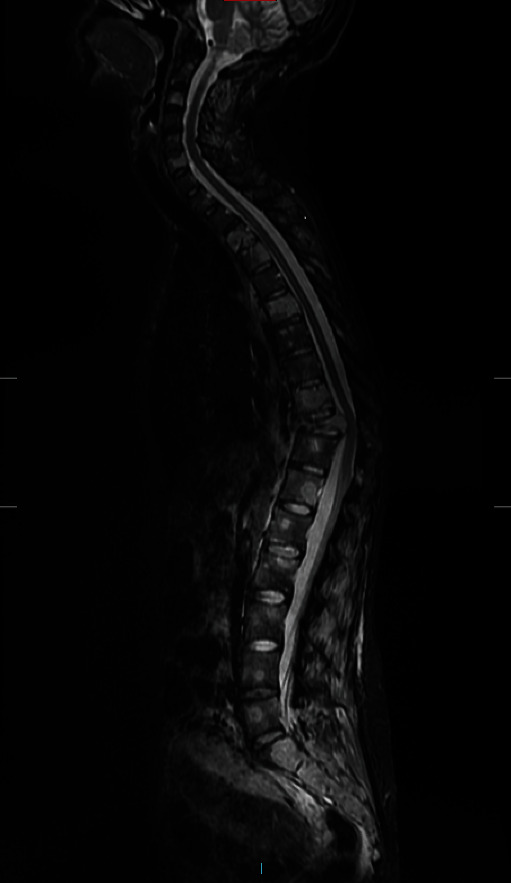
Preoperative MRI STIR.

**Figure 3:**
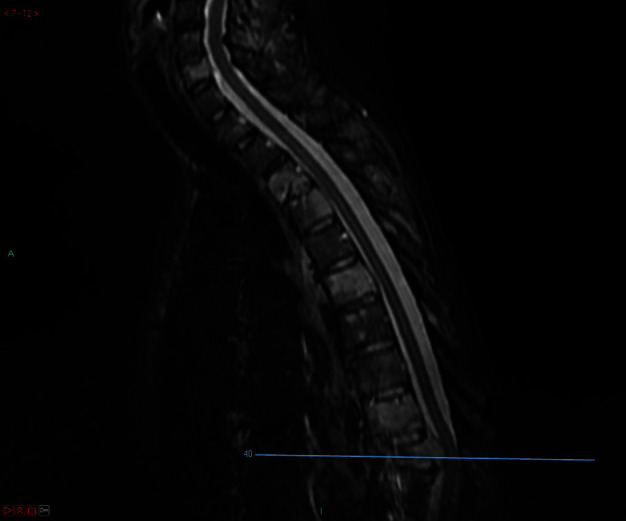
Preoperative MRI STIR (sagittal images, level of compression).

**Figure 4:**
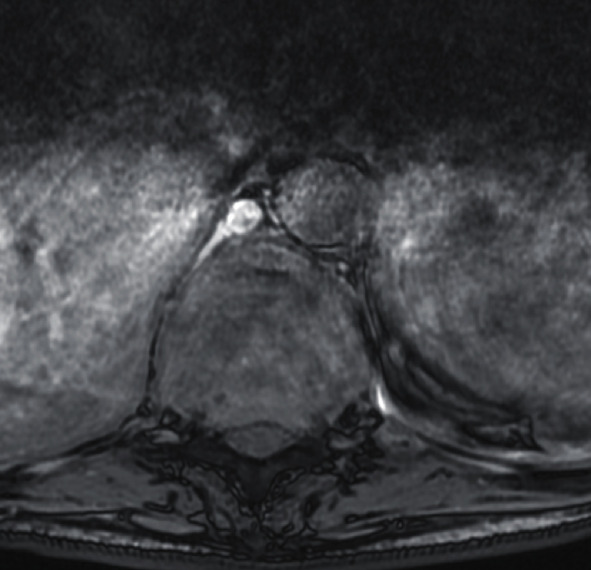
Preoperative MRI (axial image, level of compression).

Patient was 19-week pregnant at time of presentation; however, she had been complaining of severe thoracic pain (VAS score 8.9) since few weeks and initially thought this simply represented a common side effect of pregnancy. Once she started to develop weakness in lower limbs (4 out of 5 on MRC scale), her GP immediately referred her for imaging. The patient underwent a contrast-enhanced MRI (Figures 1 and Figure 2), which demonstrated metastatic spread with extensive spinal involvements extending from the cervical spine to the sacrum, with collapse of the T10 vertebral body and associated spinal cord compression (Figures 3 and Figure 4). The metastatic epidural spinal cord compression (MESCC) pathway was immediately activated, and a restaging of the disease was promptly conducted. Chest, abdomen, and pelvic CT scan and PET scan could not be safely done due to inherent risks to the foetus; hence, it was opted to proceed with chest radiograph and abdominal ultrasound. The first demonstrated left glenoid and left proximal humerus lytic lesions, and the second did not show any visceral lesions at the time. Ever since the initial diagnosis, the mother was adamant that she wanted continuation of the pregnancy and safe delivery of her baby.

In an attempt to apply predictive scoring for prognosis and survival, the Tokuhashi score was estimated to be at worst 7 and at best 9 in the absence of CT staging. The breakdown was as follows:

- Karnofsky 40% = 0 point
- Detected at least two extraspinal bone metastases = 1 point
- There were more than three vertebral body metastases = 0 point
- Major internal organ metastatic lesions were not possible to detect = 0‐2 points
- Primary malignancy was breast = 5 points
- Neurological deficit included right leg palsy (Frankel D) = 1 point

The Tomita score was also calculated, and it was estimated to be at the best 3 and at worst 7. The breakdown was as follows:

- Slow-growing breast malignancy = 1 point
- Visceral metastatic lesions were not possible to detect = 0–4 points
- Bone metastases including the spine were multiple = 2 points

Given the extensive nature of bony involvement by the neoplasm, her case was discussed with our Oncology Team. Considering that there was a reasonable prospect of disease control, the multidisciplinary team reached a consensus to offer surgery, which was accepted by the patient and her next of kin. The plan was to perform separation surgery, maintain the pregnancy, and control the disease until the delivery. Following the delivery, the oncology would implement combined chemo- and radiotherapy. An instrumented spinal fixation using pedicle screws and rods extending from T7 to L2 and focal decompression of the T10 segment was conducted. Considering the vertebral body collapse and reduction in anterior column support, we did not believe that sublaminar wires or hooks on their own can provide sufficiently rigid spinal stabilisation in comparison to pedicle screws in this situation. The patient was in positioned prone on radiolucent Allen spinal table with two chest and two pelvic bolsters allowing complete clearance of the abdomen and proper mechanical ventilation. The operation was done under general anaesthesia with a reinforced orotracheal tube. We did not use neurophysiological monitoring. Neuromonitoring was not used deliberately, as in the surgeon's experience with extradural metastasis with the aim of total thecal sac decompression neuromonitoring is not mandatory. There is evidence that intraoperative neuromonitoring is useful in resection of intradural and especially intramedullary tumours. However, that evidence is lacking when it comes to excision of extradural space-occupying lesions [4–6]. Besides, given the issues with the patient's positioning and the impact of operative time, this can be an essential factor in prolonging the duration of general anaesthesia. Surgical intervention was uneventful; the lesion was identified intraoperatively. Pedicle screw insertion was free hand; using X-ray with shielding was carried out only to localise the level of pathological fracture. Postoperatively, she recovered well, and the mechanical pain was immediately relieved. She was able to mobilise with some help thanks to improvement in motor strength in both legs. The wound healed well. The histopathological analysis confirmed that the neoplasm was oestrogen (7/8) and progesterone (8/8) receptor-positive, and negative for HER-2 gene amplification.

**Figure 5:**
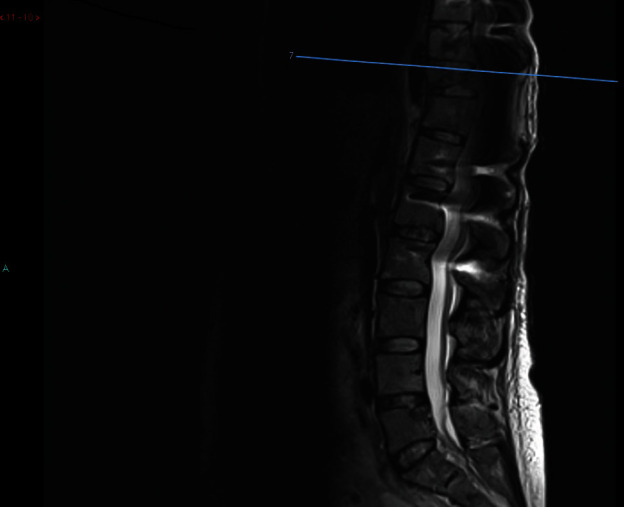
Postoperative MRI T2WI (sagittal image, showing the level of surgery).

**Figure 6:**
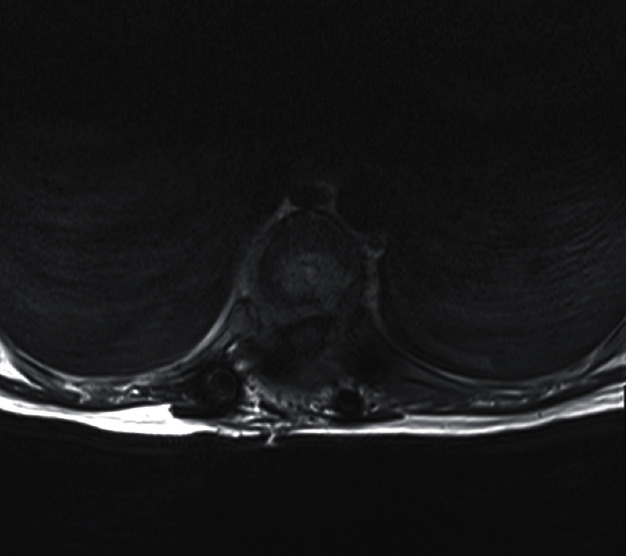
Postoperative MRI (axial image, showing the level of surgical decompression).

**Figure 7:**
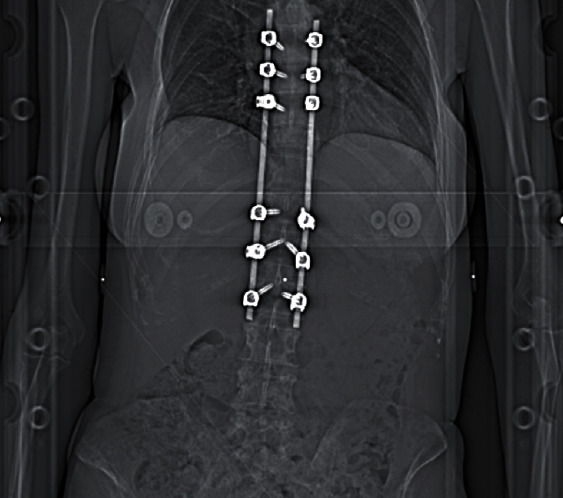
Spinal stabilisation and instrumentation construct (AP radiograph, at the time of radiotherapy).

Patient was discharged as soon as she was able to mobilise independently. An early follow up MRI performed for 1-month postoperatively (Figures 5 and Figure 6) showed a satisfactory alignment of the thoracic spine, however, the scan demonstrated some progression of involvement of T3 and T6 vertebrae without any cord compression. Two weeks later, she had the baby delivered by Caesarean section and could safely start chemotherapy and radiotherapy; the spinal stabilisation and instrumentation construct is clearly visible in Figure 7.

The long-term follow-up MRI performed for 1 year postoperatively demonstrated likely multiple liver metastases, and F18 PET scan was obtained soon after confirmed extensive bone metastatic lesion involving skull, skeleton, pelvis, and both femurs.

Following the surgery, she received nine cycles of paclitaxel weekly. The disease was defined as “stable” on MRI prior to the delivery. She had her baby delivered by Caesarean section. At that time, she underwent bilateral oophorectomy. The histology demonstrated one ovary to be infiltrated by poorly differentiated carcinoma (ER-positive (30%, moderate, 5/8), PR-positive (80%, moderate, 7/8), and HER2-positive by FISH). Following the delivery and oophorectomy, she commenced letrozole and received another course of radiotherapy (30 Gy/10 #/14 days to T7–L2 inclusive and further radiation of 8 Gy/1 # to T2–T4 inclusive). This was followed by chemotherapy (trastuzumab and denosumab). Few weeks later, palbociclib and pertuzumab were commenced. She remains on the above chemotherapeutic and endocrine agents. The disease has been under control, and the patient remains stable.

## 3. Discussion

MESCC during pregnancy carries a significant challenge for oncologists, anaesthetists, intensivists, spinal surgeons, and obstetricians. More importantly, malignancy during pregnancy causes an extremely high degree of stress to those patients and all the healthcare professionals involved in their management. In fact, these pathologies present with an overwhelming ethical dilemma and do raise a conflict between optimal maternal treatment and foetal well-being.

Overall, a systematic review on the staging and management of these challenging cases has never been conducted. Few narrative reviews have been carried out, but they all have some limitations in terms of inclusion criteria, length of follow-up, etc. Meng et al. [7] published a retrospective study on 21 pregnant women with either primary or secondary spinal lesions. Tumour types in their series included giant cell tumour (5 cases), hemangioma (5 cases), schwannoma (4 cases), eosinophilic granuloma (2 cases), neurofibroma (1 case), multiple myeloma (1 case), and with metastatic tumour (3 cases). Only two patients underwent spine surgery during pregnancy, and eight patients accepted tumour resection immediately after delivery. Pregnancy termination occurred in 5 patients, whereas the rest of the patients smoothly gave birth to healthy babies, including three premature infants. Two patients died, and two patients experienced local recurrence during follow-up. According to the authors, most of the pregnant patients with benign spinal tumours could postpone surgery after delivery. However, the authors suggested surgical treatment during pregnancy for patients who have highly malignant tumours and do experience a sharp deterioration in their neurological status. The use of chemotherapy and radiotherapy in this study was considered highly controversial. The authors considered the surgery to be much safer than radiotherapy and chemotherapy during pregnancy.

In the absence of guidelines and formal recommendations from scientific societies, we summarised here some tips for ideal management of such conditions with regard to the learning points emerged from the exemplificative case reported above.

The actual stage of the pregnancy plays a vital role in the management of these challenging cases; the option of termination of pregnancy due to progressive metastatic disease and spinal cord compression should always be offered to the patient in first and second trimesters as such approach would allow for optimal oncological treatment. This said, accepting the termination of an advanced pregnancy is always controversial and patients might often request to delay their oncological treatment despite the negative impact on disease control and overall survival. From a medicolegal perspective, all diagnostic and treatment options should be carefully discussed and documented on electronic patients' records with consideration for all related pros and cons [8]. The literature on consenting process covers this aspect extensively; this aspect has been at the centre of attention in the United Kingdom following the change in legal definition of informed consent prompted by the Supreme Court case of Montgomery vs. Lanarkshire Health Board in 2015. That landmark case for medicolegal practice shifted the focus from a traditional paternalistic model of consent towards a more patient-centred approach [9] where all options and their likely outcomes are critically discussed in lay terms to empower patients and their next of kin to an educated choice.

Related to the need to offer patients with some objective measures of prognostication upon which to guide their decision-making process, we need to consider the value of MESCC scoring systems in pregnant patients. Both the Tomita and Tokuhashi scores have been updated multiple times; however, they are based on evidence collected years ago that do not reflect the current state of the art in neurooncology practice. Furthermore, they both rely on CT staging, which is obviously not possible during pregnancy. This leads the clinicians to opt for other imaging modalities such as ultrasounds and, if needed, standard radiographs. Again, the literature does not offer any clarification on the appropriateness of advanced staging with contrast-enhanced whole-body MRI in similar cases. The practicality of long examination, side effect of sequences and contrast administration, and their risks should always be discussed with patients and radiology department [10], even more so in pregnant women, especially in their first trimester.

Spinal surgery during pregnancy is a feasible and represents a reasonable treatment modality; however, the extent of decompression and radical intent will heavily depend on clinical history and patients' presentation. Options may vary from percutaneous cement augmentation to spinal cord decompression and fixation; in some cases, excisional procedures with 360 degrees reconstruction of spinal stability can also be considered. However, several precautions are necessary during surgery in a pregnant patient as shown by our exemplificative case. The most severe obstetrical risks of surgery in pregnancy are miscarriage, preterm delivery, and foetal distress due to foetal hypoxia [11, 12]. Anaesthesiological management is critical, because maternal monitoring is essential to prevent hypoxia, hypoglycaemia, and hypotension [13, 14]. No anaesthetic drug is risk-free in pregnant women due to their hypotensive effects; however, fentanyl, remifentanil, and sevoflurane are the most commonly adopted agents due to their pharmacological profile which is known not to induce any teratogenic effect [14]. Patient positioning is also very important because safe spinal surgery can only be conducted by reducing blood loss and minimising pressure-related issues. This is particularly relevant in pregnant patients with distended uterus and diversion of a significant amount of their blood volume through the placenta [14]. As such, paddings should be carefully positioned by the surgical staff to ensure that the abdomen is free from compression, while the rest of the patients' body is well supported. Furthermore, fetouterine cardiotocography should be available and readily used [15] throughout the procedure. We carefully considered the patient's positioning in this case. We debated lateral and 3/4 prone positions as well, and their pros and cons. We consulted the literature for the use of nonconventional positioning for instrumented spinal surgery during pregnancy [16–19]. However, in this case, we used prone positioning, with appropriate abdominal padding, and avoidance of uterine compression. In our surgical experience, it allows more familiar and more comfortable orientation aiming to achieve appropriate freehand pedicle screw insertion in comparison to lateral or semiprone positions. Neurophysiological monitoring is not contraindicated in those cases; however, attention should be paid to favour fentanyl-based anaesthesia over volatile halogenated anaesthetics. Those latter impair spinal pathways by inhibiting the pyramidal activation of spinal motor neurons and synaptic transmission in the cerebral cortex. This action suppresses motor evoked potentials in a dose-dependent fashion, and although desflurane could be safely used at a MAC of 0.3-0.5, this is not enough in young pregnant women to guarantee unawareness [20]. Although the use of bispectral index (BIS) device can be utilised in cases like this, in order to monitor the depth of anaesthesia, we did not find sufficient evidence to use intraoperative neuromonitoring considering that the metastatic lesion was entirely extradural [4–6]. More importantly, due to ongoing pregnancy, we wanted to keep the duration of general anaesthesia to an absolute minimum. Intraoperative exposure to ionising radiations should obviously be minimised as much as possible, hence the need to leverage on real-time intraoperative ultrasound for guidance during resection of tumour [21–23]. One last aspect is the importance of perioperative mechanical thromboprophylaxis as shown in several clinical trials conducted in neurosurgical settings [24, 25].

The biological effects of adjuvant treatment have also to be considered carefully if a patient wishes to carry on with her pregnancy. Radiotherapy in rapidly developing foetus is incompatible with survival of the baby. Can this treatment option be ever justified during the pregnancy? The effect of radiotherapy depends on gestational age and dose of the radiotherapy used. The effects of radiotherapy in pregnancy are stochastic and deterministic. Stochastic effects occur by chance and do not have a threshold above which the effect is seen. Deterministic effects have clear causality and definite cause-effect association. The severity of the effect increases with the treatment dose [12]. According to Streffer et al. [26], the irradiation above the absorbed dose of 100 mGy increases the risk malformations in the first trimester. Otake and Schull [27] published a study, which demonstrated the high sensitivity of the CNS to radiation up to 15 weeks of gestation. And that the irradiation even of an older foetus can result in organ defects and growth restriction [26]. The concept of separation surgery has been introduced thanks to the evolution of stereotactic radiotherapy and radiosurgery. In fact, the advances in radiation therapy and the tailored use of radioenhancers promise to minimise the side effect caused by unnecessary irradiation outside the surgical site [28–30].

Chemotherapy is also contraindicated in the first trimester as it is associated with an increased risk of severe congenital malformations. During the second trimester onwards, some chemotherapeutic agents can be used [31] without increased risk of foetal malformations and significant problems in the neonatal period. The timing, dose, and number of chemotherapy cycles are crucial factors influencing the foetal outcome. Substantial risks of chemotherapy are preterm delivery, and babies being too small for gestational age. Several studies reported an increased risk of babies showing small growth for their gestational age following chemotherapy in pregnancy [32–35]. According to Cardonic and Iacobucci [34], between 7% and 17% of babies will be small for their gestational age depending on the type of cancer and chemotherapy used during pregnancy.

The issue of poor intrauterine growth leads to a substantial risk of perinatal morbidity and mortality according to the studies by Pallotto and Kilbride [36] and Sankaran and Kyle [37]. Equally, prematurity is strongly associated with neonatal morbidities and neurodevelopmental impairment. The more immature the infants are, the higher is their risk of postnatal complications and long-term impaired outcome [12].

Another aspect of significant impact in spinal surgery is the use of steroids; however, their role in pregnant patients remains controversial, especially if their prescription is needed for an extended period of time. The safety of steroid use in pregnancy with regard to preterm delivery and congenital disabilities has been the subject of fierce debate [38]. Hormonal therapy in pregnancy is equally controversial and this is particularly important in patients with breast cancers, which have benefited from this type of treatment in recent years. Breast tumours are commonly treated with tamoxifen (oestrogen antagonist). Historically, tamoxifen has been associated with foetal anomalies [39–42]. Here, the recent literature provides a positive note: according to Vandenbroucke et al. [12], most children who had in utero exposure to tangential tamoxifen are healthy, and the authors conclude that prevalence of congenital malformations as a result of tamoxifen use in pregnancy remains unknown, suggesting that the use of targeted therapy in oncology remains an important therapeutic option. While the adoption of such approach has been steadily increasing [43], the paucity of data on the safety in pregnancy is not robust enough to provide a clear evidence against Oxford Centre for Evidence-Based Medicine (OCEBM) guidelines.

Several research questions are still unanswered when it comes to the management of pregnant women with MESCC. What are the implications of delaying the curative treatment? What are the alternative treatment modalities that would keep the malignant disease under control while trying to carry on a healthy pregnancy? What is the outcome for mothers and babies if the definitive surgical and oncological treatment is delayed after delivery? What is the risk of adjuvant treatment associated with lactation? The value of this exemplificative case and narrative review lies in its contribution to shed some light on the management of pregnant patients with MESCC; however, if we are to collectively address these unanswered questions, our community needs to move forward a much closer collaboration between individual specialists including spinal surgeons, oncologists, obstetricians, and radiologists.

## 4. Summary and Conclusion

The literature and our own experience suggest that in some metastatic spinal lesions, a good outcome is possible in the second and third trimesters of pregnancy. We believe that there is a strong argument for preserving pregnancy in patients with metastatic spinal cord compression during the second and third trimesters. However, these cases require coordination of the treatment and close cooperation between surgeons, obstetricians, anaesthetists, foetal medicine specialists, and oncologists. The delivery of a healthy baby, preserving the mother's life and treating the malignant disease at the same time, represents not only a challenge but also a desirable outcome, and a very viable prospect.

If we follow a multidisciplinary approach, modern treatment modalities, adequate facilities, and emotional support for the mother and her family, it is entirely possible to maintain the pregnancy to the delivery of a healthy infant and at the same time provide adequate treatment and follow-ups for the mother. Our experience, although limited, has been overwhelmingly positive. We believe in a holistic approach to the mother and her unborn child. Last but not least, we believe that the ethical dilemmas and emotional stress for the pregnant women and her family need to be more extensively addressed; hence, in our opinion, the combined multidisciplinary approach suggested is the only one providing excellent chances of success and positive outcome for both a foetus and a mother.

## Data Availability

Data from literature search (PubMed) is available upon request.

## References

[1] Kennedy S., Yudkin P., Greenall M. (1993). Cancer in pregnancy. *European Journal of Surgical Oncology*.

[2] Murphey G. P. (1999). CA-Cancer statistics. *CA*.

[3] Weisz B., Schiff E., Lishner M. (2001). Cancer in pregnancy: maternal and fetal implications. *Human Reproduction Update*.

[4] Charalampidis A., Jiang F., Wilson J. R. F., Badhiwala J. H., Brodke D. S., Fehlings M. G. (2020). The use of intraoperative neurophysiological monitoring in spine surgery. *Global Spine Journal*.

[5] Korn A., Halevi D., Lidar Z., Biron T., Ekstein P., Constantini S. (2015). Intraoperative neurophysiological monitoring during resection of intradural extramedullary spinal cord tumors: experience with 100 cases. *Acta Neurochirurgica*.

[6] Di Perna G., Cofano F., Mantovani C. (2020). Separation surgery for metastatic epidural spinal cord compression: a qualitative review. *Journal of Bone Oncology*.

[7] Meng T., Yin H., Li Z. (2015). Therapeutic strategy and outcome of spine tumors in pregnancy. *Spine*.

[8] Dormegny L., Chibbaro S., Ganau M., Santin K. L., Proust F. (2018). Biopsying a spinal cord lesion: a diagnostic dilemma. Case report and review of literature. *Neuro-Chirurgie*.

[9] McKinnon C., Loughran D., Finn R., Coxwell-Matthewman M., Jeyaretna D. S., Williams A. P. (2018). Surgical consent practice in the UK following the Montgomery ruling: a national cross-sectional questionnaire study. *International Journal of Surgery*.

[10] Ganau M., Syrmos N. C., D'Arco F. (2017). Enhancing contrast agents and radiotracers performance through hyaluronic acid-coating in neuroradiology and nuclear medicine. *Hellenic Journal of Nuclear Medicine*.

[11] Cohen-Kerem R., Railton C., Oren D., Lishner M., Koren G. (2005). Pregnancy outcome following non-obstetric surgical intervention. *American Journal of Surgery*.

[12] Vandenbroucke T., Verheecke M., Fumagalli M., Lok C., Amant F. (2017). Effects of cancer treatment during pregnancy on fetal and child development. *The Lancet Child & Adolescent Health*.

[13] Evans S. R. T., Sarani B., Bhanot P., Feldman E. (2012). Surgery in pregnancy. *Current Problems in Surgery*.

[14] Meloni M., Serra S., Bellisano G., Syrmos N., Jeyaretna S., Ganau M. (2019). Primary gliosarcoma of the cerebellum in a young pregnant woman: management challenges and immunohistochemical features. *Case Reports in Surgery*.

[15] Amant F., Van Calsteren K., Halaska M. J. (2009). Gynecologic cancers in pregnancy. *nternational Journal of Gynecological Cancer*.

[16] Bongetta D., Versace A., De Pirro A. (2020). Positioning issues of spinal surgery during pregnancy. *World Neurosurgery*.

[17] Schnake K. J., Scholz M., Marx A., Hoffmann R., Kandziora F. (2011). Anterior, thoracoscopic-assisted reduction and stabilization of a thoracic burst fracture (T8) in a pregnant woman. *European Spine Journal*.

[18] Kaul R., Chhabra H. S., Kanagaraju V. (2016). Antepartum surgical management of Pott’s paraplegia along with maintenance of pregnancy during second trimester. *European Spine Journal*.

[19] Lenarz C. J., Wittgen C. M., Place H. M. (2009). Management of a pregnant patient with a burst fracture causing neurologic injury. *The Journal of Bone and Joint Surgery-American Volume*.

[20] Pastor J., Pulido P., López A., Sola R. G. (2010). Monitoring of motor and somatosensory systems in a 26-week pregnant woman. *Acta Neurochirurgica*.

[21] Ganau M., Syrmos N., Martin A. R., Jiang F., Fehlings M. G. (2018). Intraoperative ultrasound in spine surgery: history, current applications, future developments. *Quantitative Imaging in Medicine and Surgery*.

[22] Ganau M., Ligarotti G. K., Apostolopoulos V. (2019). Real-time intraoperative ultrasound in brain surgery: neuronavigation and use of contrast-enhanced image fusion. *Quantitative Imaging in Medicine and Surgery*.

[23] Liu T.-J., Shen F., Zhang C., Huang P.-T., Zhu Y.-J. (2018). Real-time ultrasound-MRI fusion image virtual navigation for locating intraspinal tumour in a pregnant woman. *European Spine Journal*.

[24] Chibbaro S., Cebula H., Todeschi J. (2018). Evolution of prophylaxis protocols for venous thromboembolism in neurosurgery: results from a prospective comparative study on low-molecular-weight heparin, elastic stockings, and intermittent pneumatic compression devices. *World Neurosurgery*.

[25] Ganau M., Prisco L., Cebula H. (2017). Risk of deep vein thrombosis in neurosurgery: state of the art on prophylaxis protocols and best clinical practices. *Journal of Clinical Neuroscience*.

[26] Streffer C., Shore R., Konermann G. (2003). Biological effects after prenatal irradiation (embryo and fetus). A report of the International Commission on Radiological Protection. *Annals of the ICRP*.

[27] Otake M., Schull W. J. (1984). In utero exposure to A-bomb radiation and mental retardation; a reassessment. *The British Journal of Radiology*.

[28] Rothrock R., Pennington Z., Ehresman J. (2020). Hybrid therapy for spinal metastases. *Neurosurgery Clinics of North America*.

[29] Ganau M., Foroni R. I., Gerosa M., Zivelonghi E., Longhi M., Nicolato A. (2018). Radiosurgical options in neuro-oncology: a review on current tenets and future opportunities. Part I: therapeutic strategies. *Tumori*.

[30] Ganau M., Foroni R. I., Gerosa M., Ricciardi G. K., Longhi M., Nicolato A. (2015). Radiosurgical options in neuro-oncology: a review on current tenets and future opportunities. Part II: adjuvant radiobiological tools. *Tumori Journal*.

[31] Amant F., Vandenbroucke T., Verheecke M. (2015). Pediatric outcome after maternal cancer diagnosed during pregnancy. *New England Journal of Medicine*.

[32] Van Calsteren K., Heyns L., De Smet F. (2010). Cancer during pregnancy: an analysis of 215 patients emphasizing the obstetrical and the neonatal outcomes. *Journal of Clinical Oncology*.

[33] Amant F., Van Calsteren K., Halaska M. J. (2012). Long-term cognitive and cardiac outcomes after prenatal exposure to chemotherapy in children aged 18 months or older: an observational study. *The Lancet Oncology*.

[34] Cardonick E., Iacobucci A. (2004). Use of chemotherapy during human pregnancy. *The Lancet Oncology*.

[35] Avilés A., Neri N. (2001). Hematological malignancies and pregnancy: a final report of 84 children who received chemotherapy in utero. *Clinical Lymphoma*.

[36] Pallotto E. K., Kilbride H. W. (2006). Perinatal outcome and later implications of intrauterine growth restriction. *Clinical Obstetrics and Gynecology*.

[37] Sankaran S., Kyle P. M. (2009). Aetiology and pathogenesis of IUGR. *Best Practice & Research Clinical Obstetrics & Gynaecology*.

[38] Terry A. R., Barker F. G., Leffert L., Bateman B. T., Souter I., Plotkin S. R. (2012). Outcomes of hospitalization in pregnant women with CNS neoplasms: a population-based study. *Neuro-Oncology*.

[39] Cullins S. L. (1994). Goldenhar’s syndrome associated with tamoxifen given to the mother during gestation. *JAMA: The Journal of the American Medical Association*.

[40] Tewari K., Bonebrake R. G., Asrat T., Shanberg A. M. (1997). Ambiguous genitalia in infant exposed to tamoxifen in utero. *Lancet*.

[41] Barthelmes L., Gateley C. A. (2004). Tamoxifen and pregnancy. *Breast*.

[42] Berger J. C., Clericuzio C. L. (2008). Pierre Robin sequence associated with first trimester fetal tamoxifen exposure. *American Journal of Medical Genetics. Part A*.

[43] Mendelsohn J. (2013). Personalizing oncology: perspectives and prospects. *Journal of Clinical Oncology*.

